# Combined oral low-dose cyclophosphamide endocrine therapy may improve clinical response among patients with metastatic breast cancer via Tregs in TLSs

**DOI:** 10.1038/s41598-024-64042-3

**Published:** 2024-06-11

**Authors:** Yuze Zhao, Shuo Wang, Shuzhen Lv, Xiaojun Liu, Weiping Li, Yuguang Song, Dongwen Rong, Peiming Zheng, Hongyan Huang, Huixia Zheng

**Affiliations:** 1 Department of Medical Oncology, Capital Medical University Cancer Center, Beijing Shijitan Hospital, Capital Medical University, 10 Tieyi Rd, Beijing, 100038 China; 2 Breast Department, Capital Medical University Cancer Center, Beijing Shijitan Hospital, Capital Medical University, Beijing, 100038 China; 3 Department of Pathology, First Hospital of Shanxi Medical University, Taiyuan, 030001 China; 4 Department of Pharmacology, Shanxi Medical University Fenyang College, Fenyang, 032200 China; 5 Department of Medical Oncology, First Hospital of Shanxi Medical University, Taiyuan, 030001 China

**Keywords:** Cyclophosphamide, Low dose, Regulatory T cell, Tertiary lymphoid structures, Metastatic breast cancer, Tumor microenvironment, Cancer, Drug discovery, Immunology

## Abstract

Despite limited research on refractory and/or endocrine therapy failure in elderly metastatic breast cancer (MBC) patients, a prior study showed that low-dose oral cyclophosphamide (CY) can improve the overall survival rate of MBC patients, possibly through the immunoregulation of regulatory T cells (Tregs). We preliminarily investigated the combination of endocrine therapy (ET) with oral low-dose CY as salvage therapy in elderly patients via peripheral blood regulatory T-cell analyses. In addition, we evaluated the associations of tumor tertiary lymphoid structures (TLSs) with therapeutic outcomes. HR+/HER2− advanced breast cancer patients who received low-dose CY combined with ET or ET only from April 2015 to August 2021 were enrolled in this retrospective study. The primary outcome was the clinical control rate (CCR), and the secondary outcome was progression-free survival (PFS). Circulating T lymphocyte subpopulations represented by Tregs were monitored during treatment by flow cytometry methods. TLSs wereconfirmed by hematoxylin–eosin staining of pretreatment specimens, and CD3, CD4, and Foxp3 were detected using Opal multicolor immunofluorescence. A total of 85 patients who received CY + ET and 50 patients who received ET only were enrolled, the percentage of patients who received CCR was 73% (62/85) vs. 70% (45/50), and the objective response rate (ORR) was 28% (24/85) vs. 24% (12/50). No deaths occurred during the study period. The mean PFS time was 13 vs. 11 months (*P* = 0.03). In the CY + ET group, decreases in CD4+/CD25+/Foxp3+ T cells (*P* < 0.001) were favorable for both clinical control and prolonged PFS (*P* < 0.001). Compared with patients without TLSs, those with TLSs were more likely to have better clinical control and PFS (mean time = 6 months), and a greater number of Treg cells during TLS pretreatment correlated with longer PFS (*P* = 0.043). Oral low-dose CY combined with standard ET exerts immunological effects by decreasing Treg levels to achieve improved clinical responses. Moreover, patients with TLSs might benefit more from such therapy than those without TLSs, and a high Treg cell count in TLSs before treatment predicts better therapeutic efficacy.

## Introduction

Advances in molecular pathological classification have improved the depth of precision adjuvant treatment for metastatic breast cancer (MBC). The choice of endocrine therapy depends on the expression of the hormone receptor (HR), estrogen receptor (ER) and progesterone receptor (PR), and different pharmacological mechanisms of endocrine therapy may be reciprocally replaced once disease control failure occurs. For instance, treatment in the second-line setting with single-agent nonsteroidal (anastrozole or letrozole) or steroidal (exemestane) aromatase inhibitors, fulvestrant, or tamoxifen has demonstrated some limited effects^1–5^. Therefore, the combination of endocrine treatment with tamoxifen and everolimus may effectively prolong PFS in patients who experienced endocrine therapy failure; however, 66% of these patients inevitably experience side effects^6^. More tolerable regimens are needed clinically.

Some controversial data support the tangible efficacy of immunotherapy in MBC because the naturally hormone-dependent cell cycle of breast cancer cells leads to insensitivity to immunotherapy. The binding of the MHC-I antigen to the T-cell receptor (TCR) induces intra- and intercellular immune signal transduction and subsequent antitumor immunity and clinical responses. Relatively low or absent expression of TCR has been observed in HR-expressing breast cancer but not in triple-negative breast cancer (TNBC), and encouraging clinical responses have been reported when immune checkpoint inhibitors(ICIs) have been used in such MBC populations. Treg cells play an important role in blocking antitumor immunity^7,8^, and the selective depletion of Treg cells might restore or enhance the biological function of ICIs. Low-dose CY selectively targets Tregs in part through the inhibition of miRNA-142-3p by Foxp3. Some studies have reported the role of low-dose cyclophosphamide in the treatment of MBC^9–12^, but little attention has been given to elucidating the relationship between Treg cells in peripheral blood and patient prognosis after combination with endocrine therapy.

Tertiary lymphoid structures (TLSs) are a type of heterotopic lymphoid tissue similar to secondary lymphoid organs, such as lymph nodes and the spleen. The importance of tissue-infiltrating lymphocytes (TILs) in breast tumor development and therapeutic responses is widely accepted, but the mechanisms underlying their effects and alterations in lymphocyte composition during tumor progression remain poorly understood^13^. TILs and tertiary lymphoid cells with specialized high endothelial venules (HEVs) have predictive and prognostic significance in breast cancer^14–16^. Emerging data support a role for the host immune response to invasive breast cancer, specifically the infiltration of the tumor microenvironment by lymphocytes and myeloid cells, as well as the prognostic and therapeutic benefit of TLSs^17^. For example, the density of TILs in breast cancer patients has been found to be related to both the response to neoadjuvant chemotherapy and the prognosis of patients who receive adjuvant chemotherapy^18^. The general consideration of immune checkpoint inhibitor (ICI) administration is currently strongly dependent on the expression of PD1 and/or PDL1 molecules, which are closely related to prognosis. A recent study revealed that high PD-1 expression in intratumoral TILs is associated with increased overall survival (OS) and disease-free survival (DFS)^19^. In general, PD-L1 expression in breast tumors is associated with TLSs^20^, and CD8^+^ T-cell infiltration is typically associated with better clinical outcomes^15^.

Therefore, we performed this retrospective study to evaluate the clinical and immunomodulatory effects of low-dose oral cyclophosphamide combined with endocrine therapy on Treg cells in elderly patients. We also profiled the composition of TLSs, detected the expression of CD3, CD4, and Foxp3 on TILs and associated the data obtained with prognostic factors and survival.

## Patients and methods

### Study design and patients

A retrospective analysis was performed on postmenopausal women with histologically confirmed MBC who were ER− and/or PR+. Since the overall survival currently does not significantly differ between patients with metastatic receptor-positive breast cancer treated with fulvestrant or letrozole^21^, we enrolled 135 patients who received cyclophosphamide combined with letrozole/fulvestrant or letrozole/fulvestrant treatment only after previous therapy failure between April 2015 and August 2021 at our clinic. Eighty-seven of the postmenopausal women were at least 65 years of age; 48 women were younger than 65 years of age, had cessation of regular menses for at least 12 consecutive months with no alternative pathologic or physiological cause and had serum levels of follicle-stimulating hormone and estradiol in the postmenopausal range. The exclusion criteria included HER2 positivity, combined treatment with cyclin-dependent kinase (CDK) 4 and 6 inhibitors and treatment with a mammalian target of rapamycin (mTOR) inhibitor. This retrospective study was approved by the Shanxi Medical University Institutional Ethics Committee of (No. 2018034), and the requirement to obtain informed written consent was waived. Written informed consent for the research was waived due to the retrospective nature of this study. All procedures performed in this study involving human participants were in accordance with the Declaration of Helsinki (as revised in 2013).

### Treatment

In this study, 85 of 135 patients received cyclophosphamide (50 mg oral daily) combined with endocrine therapy (CY + ET), 50 patients received cyclophosphamide plus letrozole, and 35 patients received cyclophosphamide plus fulvestrant. Fifty of 135 patients received endocrine therapy (ET) only, 21 patients received letrozole and 29 patients received fulvestrant. Cyclophosphamide (CTX, 50 mg) was administered orallydaily, and letrozole (2.5 mg) was administered orally daily. Fulvestrant (0.25 g/needle) was injected into the left and right hips monthly, and 0.5 g was administered 2 weeks after the first injection. Peripheral blood for T-cell phenotype analysis was collected from each patient 1 day before and 2 months after taking cyclophosphamide. Each patient received at least 2 cycles of cyclophosphamide-based treatment, followed by monthly clinical examinations.

### Response and safety assessments

Response to treatment was assessed using the RECIST version 1.1 criteria, as follows: complete response (CR), all target lesions disappeared; partial response (PR), the sum of the longest diameters of the target lesions was reduced by at least 30% compared with the baseline state; stable disease (SD), between partial remission and disease progression; and progressive disease (PD), the sum of the longest diameters of the target lesions was increased by 20% compared to the sum of the longest diameters of the smallest target lesions recorded after the start of treatment, or one or more new lesions were present. The second objective was to estimate PFS according to RECIST version 1.1. The third objective was to estimate the mean PFS, objective response rate (ORR, CR + PR), and clinical control rate (CCR, CR + PR + SD) to evaluate the safety of the regimen and to summarize the distributions of immune subsets at baseline and changes during treatment.

Adverse events were evaluated using the National Cancer Institute Common Toxicity Criteria (CTCAE), Version 4.0.

### Flow cytometric analysis of the peripheral blood T-cell phenotype

Peripheral venous blood was obtained from each patient, and T-cell subsets were enumerated by flow cytometry following the instructions of Beckman-Coulter. The primary antibodies used included anti-CD4-FITC, anti-CD8-PE, anti-CD3-PerCP (Becton–Dickinson), anti-CD4-FITC (Beckman-Coulter), anti-CD25-PE (Beckman-Coulter), anti-CD28-FITC (Beckman-Coulter), anti-Foxp3-FITC (Beckman-Coulter) antibodies, anti-CD8-PE (Beckman-Coulter), and anti-CD3-FITC (Beckman-Coulter). One hundred microliters of blood was incubated in the dark with the primary antibody at 4 °C for 15 min. After hemolysis for 10 min, the samples were centrifuged at 1500 rpm for 10 min at room temperature, washed twice in PBS and subjected to flow cytometric analysis using an FC500 (Beckman-Coulter) with CXP analysis software (Beckman-Coulter). Lymphocyte populations are reported as percentages of the total population.

### Human tissues and histological evaluation

All specimens were obtained by recurrent breast tumor core needle biopsy after the failure of first-line endocrine therapy; the specimens were formalin-fixed, paraffin-embedded, and well preserved. Complete clinical and pathological data and follow-up records were available for all patients. TLSs were diagnosed by pathologists according to H&E staining. A TLS is defined histologically as an aggregation of lymphocytes containing blood vessels with clear HEV characteristics (plump and cuboidal endothelial cells)^22^.

### Immunofluorescence staining and antibodies

Paraffin-embedded samples fixed in 10% neutral formalin were cut into 5-µm-thick sections. H&E staining and Opal polychromatic immunofluorescence analyses were subsequently performed. Two pathologists who were blinded to the patients’ clinicopathological characteristics independently evaluated the films and manually counted the number of TLSs.

The multiplex antibodies and materials used for multicolor immunofluorescence were Panel 1 (for lymphocyte subsets) :anti-CD4 (ab133616, Abcam, USA, 1:500, Opal 480), anti-CD3 (ab237707, Abcam, USA, 1:500, Opal 690), anti-FoxP3 (98,377, Cell Signaling, USA, 1:100, Opal 570) , Panel 2 (for TLSs) : anti-CD103 (ab224202, Abcam, USA, 1:400, Opal 620), anti-CD20 (48750S, Cell Signaling, USA, 1:300, Opal 690), anti-CD3 (ab 237707, Abcam, USA, 1:500, Opal 520), anti-CXCL13 (ab246518, Abcam, USA, 1:1000, Opal 570) and an Opal 4-color fluorescence staining kit (lot number: 2306158, Boston, MA, USA). Sections were deparaffinized in xylene and rehydrated in a series of graded alcohols. In a serial fashion, each antigen was labeled by distinct fluorophores.

### Digital image acquisition and analysis

A Vectra 2.0 (PerkinElmer, Waltham, MA, USA) slide scanning system was used to scan the fluorescently labeled slides using a Zeiss Axio Imager Z2 upright epifluorescence microscope. The cell counts, mean intensity, percentage of positive cells among the total number of cells (positive cells/all nucleated cells), and positive cell density were measured using Form 2.1 analysis software (PerkinElmer).

### Statistical methods

Lymphocyte subset analysis variables were expressed as the mean standard deviation (SD) and were compared using a two-tailed unpaired Student’s t test. Binary logistic regression was used to identify risk factors associated with disease remission. The results are reported as hazard ratios (HRs) and 95% confidence intervals (CIs). An HR > 1 indicated elevated risk with respect to the reference category. The fluorescence intensity of each pixel reflected the expression level of the marker corresponding to the fluorescence value, and we calculated the amount of each marker expressed by each cell in the representative field of view. The number of cells positive for the specific marker and the proportion of the total number of cells were also calculated. All data are expressed as the mean ± SEM. Survival was estimated for each group using the Kaplan–Meier method, and the data were considered separately and compared statistically using the log-rank test. All the statistical analyses were carried out using SPSS software (Statistical Package for the Social Science, version 15.0, SPSS Inc., Chicago, IL, USA). Statistical analysis was conducted with GraphPad Prism software; *P* values were calculated using the unpaired double-tailed *t* test (*P < 0.05; **P < 0.001; ***P < 0.0001; Ns, not significant).

## Results

### Patients

A total of 135 female patients were considered for this study. The baseline patient characteristics are summarized in Table 1. The average age of the CY + ET population was significantly greater (63 years) thanthat of the ET group (61 years, *P* > 0.05). We selected an HR+/HER2− population, and all patients were ER positive; some of them had simultaneous PR, but only a small portion of them had simultaneous ER and PR expression Most of the enrolled patients developed lung and bone metastases. The enrolled patients were elderly patients with advanced metastatic breast cancer; a few of them had undergone chemotherapy, and all of them had undergone endocrine therapy prior to enrollment. Letrozole/fulvestrant was not applied prior to endocrine therapy. The ECOG score of the patients in the study was 0 or 1 point, indicating a fair general condition.

**Table 1:** Demographics and baseline characteristics of patients.

Variable	ET		CY + ET	
	Total	Percentage (%)	Total	Percentage (%)
Case, n	50	100.00	85	100.00
Sex				
Female	50	100.00	85	100.00
Age (mean)	61		63	
≥ 65	32	64	55	65.00
< 65	18	36	30	35.00
Receptor expression				
ER+	50	100.00	85	100.00
PR+	17	34	29	35.00
HER2−	50	100.00	85	100.00
Numbers of metastasis organ				
1	5	10.00	10	12.00
2	26	52.00	45	53.00
> 2	19	38.00	30	35.00
Previous adjuvant chemotherapy				
Yes	24	48.00	38	45.00
No	26	52.00	47	55.00
Previous adjuvant endocrinotherapy				
Yes	22	44.00	35	41.00
No	28	56.00	50	59.00
ECOG-PS				
0	27	54.00	45	53.00
1	23	46.00	40	47.00
Therapy				
CY + Letrozole	–	–	50	59.00
CY + Fulvestrant	–	–	35	41.00
Letrozole	21	42.00	–	–
Fulvestrant	29	58.00	–	–
Disease control				
CR + PR + SD	35	70.00	62	73.00
PD	15	30.00	23	27.06

### Treatment effect

**Figure 1:**
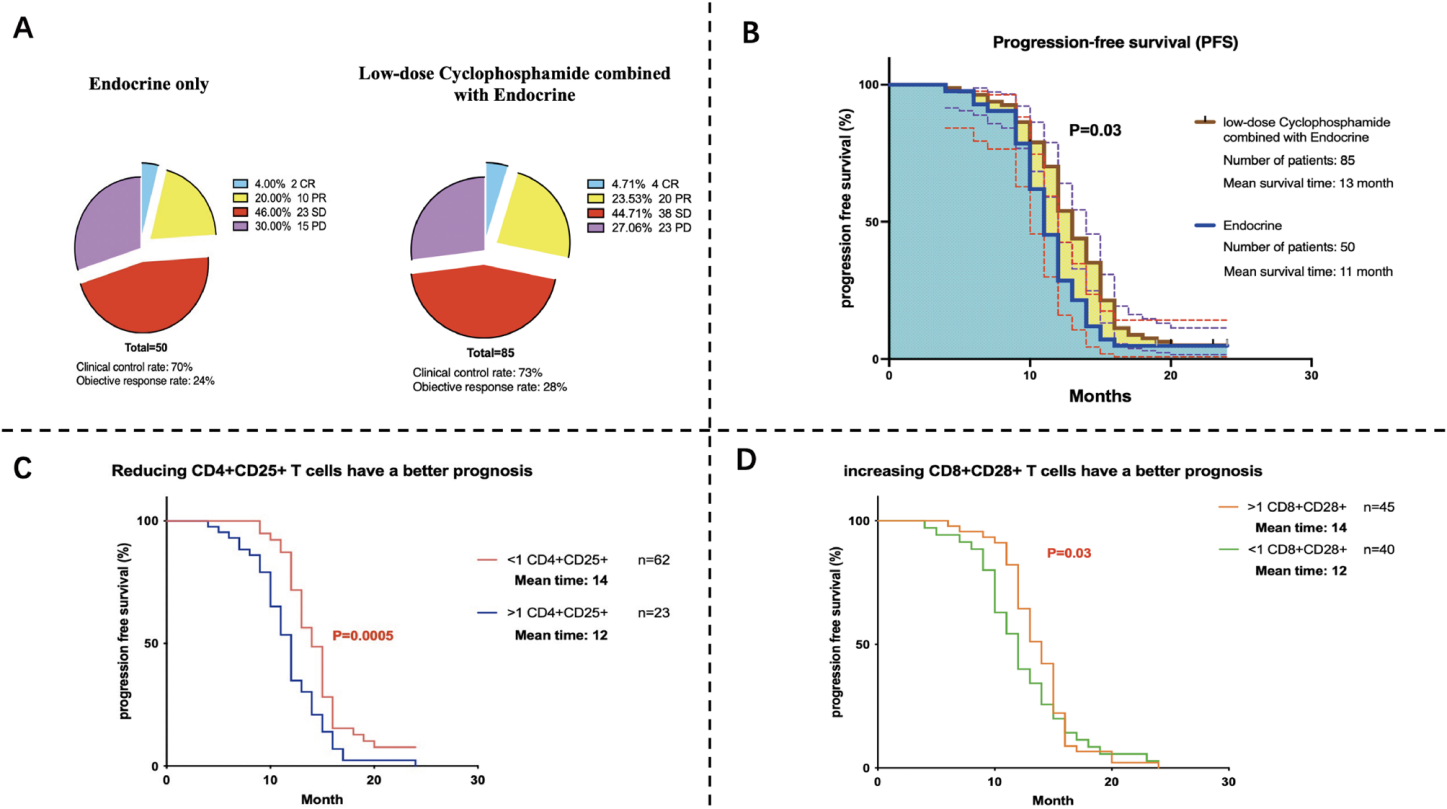
Prognosis of HR+ breast cancer patients after low-dose CY + ET treatment. (**A**) Disease control in patients after low-dose CY + ET treatment. (**B**) PFS of patients after low-dose CY + ET treatment. (**C**) Patients with reduced peripheral blood Treg cell counts after low-dose CY + ET treatment have a better prognosis. (**D**) Patients with increased CD8+CD28+ T-cell counts in peripheral blood after low-dose CY + ET treatment had a better prognosis.

Based on previously reported criteria, the therapeutic response of CY + ET patients at 2 months was assessed as 4 CR, 20 PR, 38 SD and 23 PD cases patients, for a CCR of 73% (62/85) and an ORR of 28% (24/85). The ET group, included 2 CR, 10 PR, 23 SD and 15 PD cases, patients, and the CCR and ORR were 70% (35/50) and 24% (12/50), respectively (Fig. 1A). No patient died during the study period.

The mean PFS times of the CY + ET and ET groups were 13 months and 11 months, respectively (*P* = 0.03) (Fig. 1B).

### Treatment toxicity

Overall, the two regimens were both very well tolerated, and grade 4–5 adverse events did not occur. In the CY + ET group, the most frequent relevant adverse events recorded were bone-related events and digestive events; 11 (12.94%) adverse events occurred among the patients, most of which (8/11;73%) were mild or moderate in severity (grade 1 or 2), except3 patients (3.5%) who had grade 3 adverse events: leukocyte decrease (n = 1), urinary tract infection (n = 1), and nausea and vomiting ( n = 1). In the ET group, 3 (6%) adverse events were observed: grade 2 adverse events were headache (n = 1, 2%) and palpitations (n = 2, 4%). No patients experienced treatment interruption or delay in either arm.

### Factors associated with the efficacy of low-dose cyclophosphamide combined with endocrine therapy

Binary logistic regression models were used to quantify factors associated with CY + ET clinical control. After adjusting for competing risk factors, the number of metastatic sites (*P* = 0.017) remained an independent factor associated with therapeutic efficacy. However, other factors, such as ECOG-PS score (*P* = 0.971), receptor expression (*P* = 0.093), combination treatment (fulvestrant or letrozole) (*P* = 0.073) and duration of therapy (*P* = 0.113), had no effect on treatment outcomes. The details are shown in Table 2. Fulvestrant and letrozole show no significant difference in the overall survival of recipient-positive breast cancer patients^21^, and our results suggest that fulvestrant and letrozole had no effect on the prognosis of our enrolled patients. For subsequent analysis, the patients were followed together as an endocrine combined low-dose cyclophosphamide group.

**Table 2:** Binary logistics regression analysis of patients’ demographic and clinical characteristics.

Variables	Remission	
	HR (95% CI)	*P* value
ECOG-PS: 2	0.872 (0.684–1.327)	0.971
Difference combined therapy	1.125 (0.927–1.352)	0.073
Difference period therapy	1.188 (0.354–2.1)	0.113
Receptor expression	1.227 (0.248–2.397)	0.093
Number of metastasis organs	0.548 (0.275–0.977)	0.017

### Phenotypic analysis of peripheral blood immune cells

**Figure 2:**
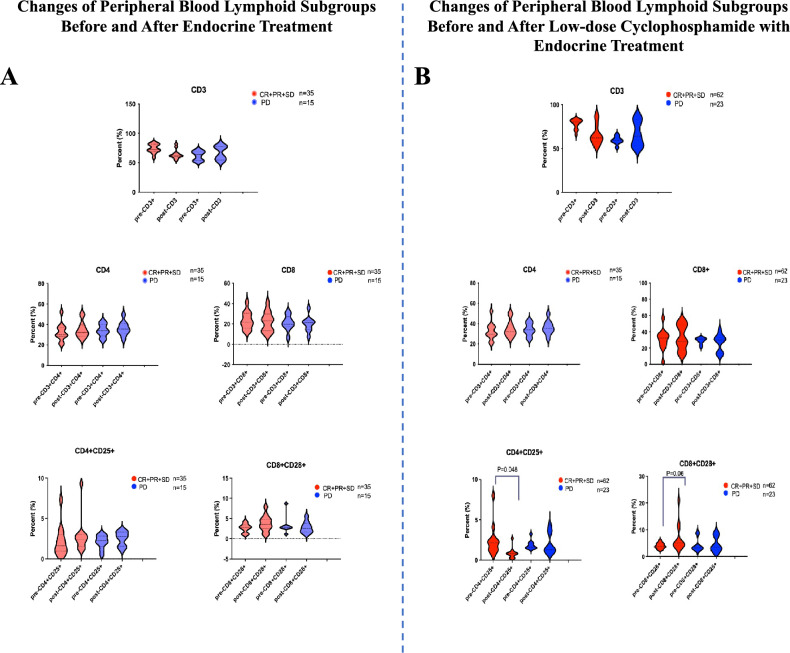
Changes in the T lymphocyte subset and plasma cytokines in peripheral blood after low-dose CY + ET treatment. (**A**) Changes in peripheral blood lymphoid subgroups before and after low-dose CY + ET treatment. (**B**) Changes in peripheral blood cytokines before and after low-dose CY + ET treatment.

Peripheral blood samples were collected from patients before and after 2 months of CY + ET or ET to extract PBMCs, and the proportion of each lymphocyte subset was analyzed by flow cytometry. Specifically, CD4+CD25+Foxp3+ T cells were defined as Treg cells and CD3+CD8+CD28+ T cells were defined as CTLs. The total numbers of CD3+, CD4+, or CD8+ T cells, Tregs or CTLs did not markedly differ before or after ET (Fig. 2A). In comparison, in the CY + ET group, total CD3 + T cells were compared between the CCR group, and the PD group, but the results revealed no significant differences. Moreover, the total CD4+ or total CD8+ T cells did not markedly differ after CY + ET. However, the proportion of Tregs in the population sensitive to CY + ET was markedly reduced after treatment (*P* = 0.048), although it was not markedly changed in patients who were insensitive, and the proportion of CTLs in the population was larger than that in the treatment-sensitive population (*P* = 0.06) but remained unchanged in treatment-insensitive patients before and after treatment (Fig. 2B).

Based on the above experimental results, the ratios of Treg cell counts before and after treatment i were calculated in all patients; a Treg ratio > 1 indicated an increase in Treg count after treatment, whereas a Treg ratio < 1 suggested a decrease in Treg count after treatment. The PFS of patients in the CY + ET arm was determined, The PFS of patients with decreased Treg cell counts was longer than that of patientswith elevated Treg cell counts, with a mean PFS of 14 months (*P* < 0.001), as indicated in Fig. 1C. Regarding the effect of CTL changes on PFS, the PFS of patients with elevated CTLs after CY + ET was longer than that of patients with decreased CTLs, but the difference was not remarkable (Fig. 1D; *P* = 0.03).

### *Effect of TLSs on the composition of T lymphoid subpopulations in CY* + *ET arm*

**Figure 3:**
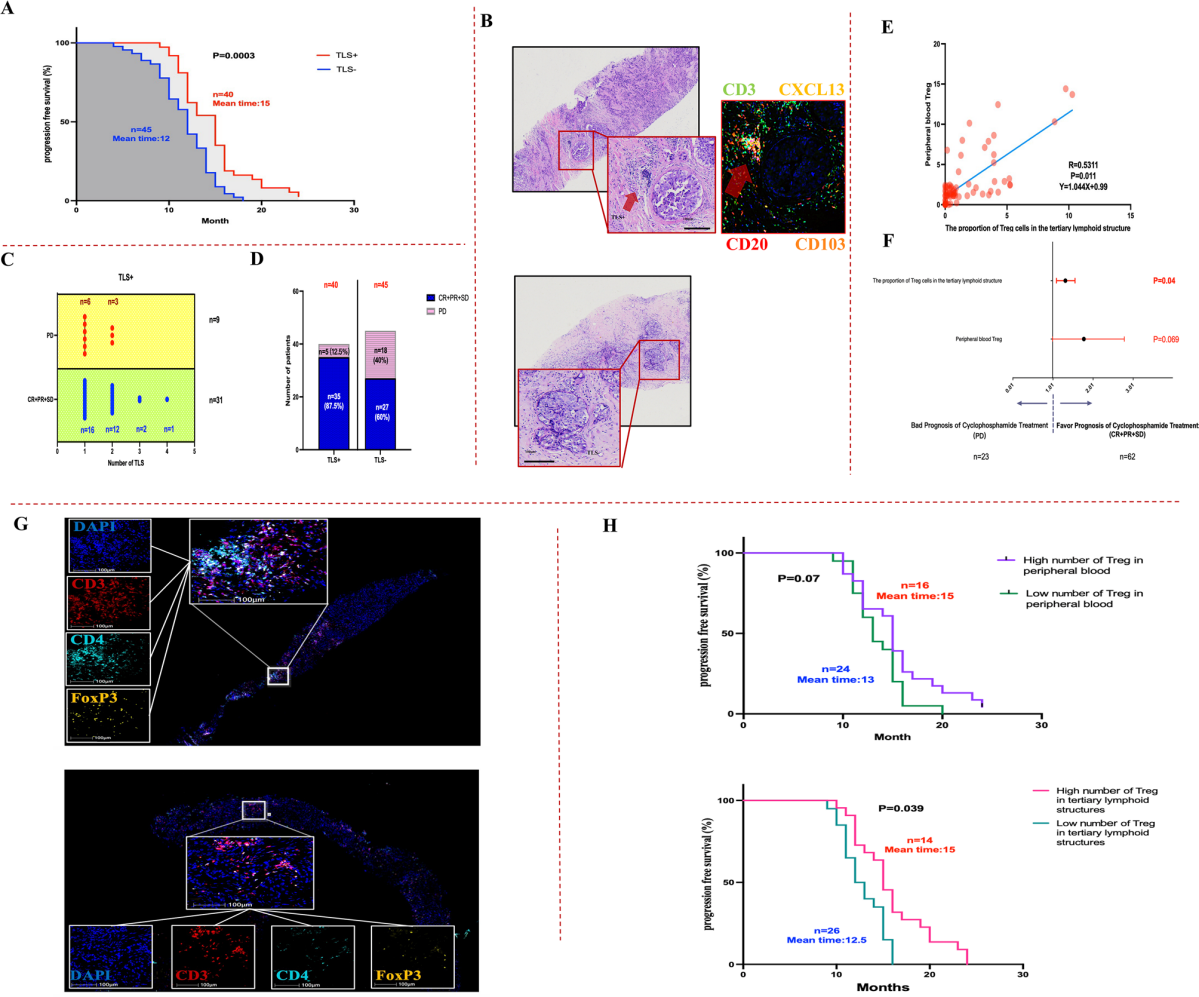
The presence of TLSs and the number of Tregs in TLSs before treatment affect prognosis. (**A**) Patients who received TLS pretreatment had a more favorable prognosis. (**B**) H&E staining was used to observe TLSs. **(C**) The number of TLSs in patients with different prognoses. (**D**) The presence of TLSs significantly differed in patients with different prognoses. (**E**) The presence of Treg cells in TLSs and peripheral blood was positively correlated. (**F**) The number of Tregs in TLSs before treatment affected prognosis. (**G**) The difference in the number of Treg cells among TLSs was evaluated by multicolor immunofluorescence staining. (**H**) Patients with higher numbers of Tregs in TLSs had longer PFS after low-dose CY + ET treatment.

In the CY + ET arm, to compare the effect of TLSs on the immune microenvironment in the CCR group and PD group , TLS diagnosis was initially confirmed by hematoxylin–eosin staining of pretreatment specimens, and then CD3, CD20, CD103, and CXCL13 were detected using Opal multicolor immunofluorescence. (Fig. 3B). TLSs were detected in 47% of the total patients. PFS in the TLS-positive (TLS+, n ≥ 1) and TLS-negative (TLS-) subgroups was 15 months and 12 months, respectively (*P* < 0.001) (Fig. 3A). Next, we compared the proportions of TLS-containing patients in the different prognostic subgroups. The TLS-containing ratio was significantly greater in the sensitive group (CCR) (87.5%) than in the nonsensitive group (PD) (12.5%) (Fig. 3D). Evaluating the number of TLSs revealed that the percentages of patients with more than one TLS in the sensitive group and nonsensitive group were 50% (31/62) and 39% (9/14), respectively, and the largest number of TLSs in the CCR and PD patients was 4 and 2, respectively (Fig. 3C).

Furthermore, we used multiple-target immunofluorescence staining to analyze the composition of different lymphocyte subsets. In the CCR group and PD group, the total number of CD3^+^ CD4+FoxP3 T cells in TLS-containing patients was significantly greater than that in patients without TLSs , the proportion of Treg cells in tumor TLSs was quantitatively analyzed by a fluorescence-based assay to study the effect of Treg cells on the immune microenvironment (Fig. 3G). Because all tumor biopsy specimens were obtained before CY + ET treatment, we performed a correlation analysis between the proportion of Treg cells in TLSs and the ratio of Treg cells to mononuclear cells in peripheral blood (Fig. 3E) and found a positive correlation (*P* = 0.011, R = 0.5311, Y = 1.044X + 0.99). This finding indicates that an increase in the proportion of Treg cells in TLSs is usually accompanied by an increase in the proportion of Treg cells in peripheral blood. Therefore, we hypothesized that the number of Treg cells in TLSs can predict the effect of CY + ET treatment. First, multivariable logistic regression analysis was used to assess whether the number of Treg cells in TLSs and peripheral blood before treatment are predictors of clinical response (CCR/PD). As depicted in Fig. 3F, both in TLSs and peripheral blood, higher numbers of Treg cells predicted a better prognosis; this trend was more significant in TLSs. Furthermore, we stratified the number of Treg cells in TLSs and peripheral blood to evaluate the correlation between the number of Treg cells and PFS. Treg cell positivity in TLSs greater than 1.6 was defined as high number; Treg cell positivity in TLSs less than 1.6 was defined as low number. A statistically significant relationship was detected between a greater proportion of Treg cells in TLSs and a better treatment response (*P* = 0.04). In addition, PFS tended to be longer in patients with a greater proportion of Treg cells (mean time: 15 vs. 12.5 months, *P* = 0.039) (Fig. 3H).

## Discussion

At present, treatment options for elderly breast cancer patients with positive receptors are lacking and are dominated by endocrine therapy; chemotherapy is employed in the presence of resistance and progression^23^. However, the overall therapeutic effect is poor, and the clinical response rate in elderly breast cancer patients receiving combined exemestane and everolimus is only 12.6%^24^. Moreover, chemotherapeutics frequently induce myelosuppression and gastrointestinal reactions in elderly patients and cause great suffering to patients^25^. In our research, the PFS of patients in the ET arm was 11 months which was similar to that reported in previous studies^26,27^.

We noticed that, the low-dose CY + ET group led to showed a favorable therapeutic effect for elderly breast cancer patients compared with the only ET group, with a clinical control rate after treatment of as high as 73%, an objective response rate of 28%, and a median PFS of 13 months, thus achieving an excellent therapeutic effect. Moreover, compared with those in the ET group, no more grade 4–5 adverse events were reported in any patients during treatment. In our research, the final therapeutic effect was not affected by various prior treatments or different endocrine drugs. Nevertheless, the occurrence of multiple metastases in some patients generally predicts poor treatment outcomes. At the late stage of the disease, multiple antitumor-suppressing immunocytes function cooperatively, such as myeloid-derived suppressor cells (MDSCs) and M2 cells, but these cells are not sufficiently sensitive to cyclophosphamide and cannot be effectively controlled, possibly leading to poor prognosis^28^.

Strategies to relieve antitumor immunosuppression have been extensively applied in tumor treatment and attained, favorable clinical effects. Among these strategies, immune checkpoint inhibitors can effectively block antitumor suppression pathways, such as PD1-PDL1, enhancing the killing effect of T cells. In our previous study, we amplified peripheral blood mononuclear cells, increased the directional amplification of cytokines, cultured tumor-killing DC-CIK cells and transfused them back into patients, which also resulted in favorable clinical effects^29–32^. In healthy donors, Tregs constitute approximately 2–4% of circulating CD4+ T cells^33^. Tregs have physiologicalimmunosuppressive functions but also play a fundamental role in tumor immune evasion^34^. In cancer patients, Treg numbers are increased in the peripheral blood and are prevalent in the tumor itself as well as in tumor-draining lymph nodes, which leads to a worse prognosis^35,36^. Strategies to deplete Tregs (at least transiently) in patients have been intensely studied. Low-dose CY selectively affects Tregs^37–39^. Our study was designed to observe the effect of low-dose CY treatment on changes in the ratio of Treg cells to mononuclear cells, and baseline comparisons were also made. We found no statistically significant difference in the proportion of Tregs between the two groups at baseline, before enrollment, which indicated that the change in the proportion of Tregs had a greater impact on the prognosis, in the other words, the reduction of the proportion of Tregs caused by cyclophosphamide can effectively prolong the survival time of patients. In addition, we discovered through flow cytometry that if Treg cells in patients were suppressed successfully after taking CY, the corresponding CTL cell number was elevated; this change would ultimately reduce antitumor suppression and increase the tumor killing effect, prolonging survival time. Moreover, the proportion of Treg cells decreased after oral administration of low-dose CY combined with endocrine therapy, and the PFS of these patients was markedly longer than that of patients with an increased proportion of Treg cells (Supplementary information).

Mature TLSs consist of numerous relevant immune cells, such as B-cell follicles surrounded by a network of follicular helper T cells and follicular dendritic cells (DCs) and T-cell-enriched regions with dendritic cells (DCs), and emphasize the spatial proximity of specialized subsets of immune cells within TLSs^40^. A recent study revealed that TLS presence was strongly associated with positive immunoreactivity and favorable prognosis in most types of solid tumors^41^. TLSs have been studied mainly in TNBC and HER2 + BC molecular subtypes and are associated with improved clinical outcomes^13^. Most studies have shown that the presence of TLSs is negatively associated with the expression of ERs^42^. We evaluated TLSs in patients who received CY + ET, as a greater presence of TLSs is closely related to a favorable TME and a positive response to multiple measures, including chemotherapy and immunotherapy. In our study, the proportion of TLS + cells in HR+/HER2− BC patients was 47.1%, which is similar to that reported in previous research (51.71%)^43^. Significant variation between the TLS + subgroup and TLS − subgroup receiving the same CY + ET treatment had a significant positive impact on BC progression-free survival (PFS) and therapeutic efficacy. .

In a recent study, Li et al*.* used the “immunoscore” determined by intratumoral immune cells to grade TLSs, which might provide a meaningful and convincing prognostic tool for oral squamous cell carcinoma. Furthermore, they found that TLSs combined with CD8+ T cells provide better prognosis prediction, similar to our conclusion in BC^14^.

According to previous studies, TLSs play important roles in different cancers (lung cancer, breast cancer, and melanoma)^44^, and because TLSs are adjacent to tumor tissue, TLSs have been investigated for their ability to regulate the TME^45^. Low-dose CY has been reported to induce antitumor immune reactions by reducing Tregs by more than 40% ^46^. To examine whether low-dose CY also causes a significant reduction in the number of TLS Tregs, which is related to the therapeutic effect, we collected recurrent breast tumor core needle biopsy specimens and identified TLS-positive or TLS-negative cells. We investigated whether patients with TLSs had longer PFS after low-dose CY + ET treatment. The proportion of TLS+ patients in the DC group was significantly greater than that in the PD group, and in terms of the TLS+ cell count, the ratio of patients with more than one TLS+ cell in the DC group was greaterthan that in the PD group. These results suggest that low-dose CY + ET treatment might regulate the TME to improve the therapeutic response related to TLSs. Recently, TLSs have been studied extensively, and multiple-target immunofluorescence staining has been used to analyze the count and location of various immune cells and to explore the function of TLSs by determining the composition of different lymphocyte subsets in TLSs^47,48^. Therefore, to investigate whether Tregs in TLSs can relieve immunosuppression and prolong PFS, we used multiple-target immunofluorescence staining to assess the number and ratio of Tregs in TLSs. We further found by correlation analysis that the proportions of Treg cells in TLSs and peripheral blood were positively correlated, suggesting that Tregs in TLSs reflect the state of Tregs in the peripheral blood, thus providing insight into general antitumor immunity. In addition, we used multivariable logistic regression analysis and showed that compared with peripheral blood, the change in the Treg count in TLSs adjacent to breast tumors more accurately predicted the effect of low-dose CY + ET treatment. Finally, we defined the cutoff point as the median number, classified high and low numberof Tregs in TLSs and peripheral blood, respectively, and discovered that Tregs in TLSs are affected by low-dose CY + ET therapy. According to our results, although Tregs in TLSs have immunosuppressive functions, the suppression caused by the increase in Tregs could be reversed by low-dose CY + ET therapy, which improved the immunological function of TLSs. Since TLSs are adjacent to tumors, reducing the number of Tregs in TLSs could more effectively enhance the role of TLSs in regulating antitumor immunity and lead to improved prognosis.

## Conclusions

The appropriate TME is well known to be a critical factor in positive clinical responses. We discovered that the oral administration of low-dose CY combined with endocrine therapy in elderly patients with metastatic HR+/HER2− breast cancer suppressed the Treg cell count and strongly correlated with the presence of TLSs. The Treg cell count in TLSs was positively correlated with that in peripheral blood and predicted therapeutic efficacy before treatment. Based on the above results and discussion, compared with ET alone, CY + ET plays an important immunomodulatory role in HR+/HER2-BC, leading to a more favorable clinical therapeutic effect and a better prognosis.

### Supplementary Information

Supplementary Information.

## Data Availability

All supporting data included within the main article are available from the corresponding author (HZ), upon reasonable request.

## References

[1] Rose C, Vtoraya O, Pluzanska A, Davidson N, Gershanovich M, Thomas R (2003). An open randomised trial of second-line endocrine therapy in advanced breast cancer comparison of the aromatase inhibitors letrozole and anastrozole. Eur. J. Cancer.

[2] Chia S, Gradishar W, Mauriac L, Bines J, Amant F, Federico M (2008). Double-blind, randomized placebo controlled trial of fulvestrant compared with exemestane after prior nonsteroidal aromatase inhibitor therapy in postmenopausal women with hormone receptor-positive, advanced breast cancer: results from EFECT. J. Clin. Oncol..

[3] Di Leo A, Jerusalem G, Petruzelka L, Torres R, Bondarenko IN, Khasanov R (2010). Results of the CONFIRM phase III trial comparing fulvestrant 250 mg with fulvestrant 500 mg in postmenopausal women with estrogen receptor-positive advanced breast cancer. J. Clin. Oncol..

[4] Di Leo A, Jerusalem G, Petruzelka L, Torres R, Bondarenko IN, Khasanov R (2014). Final overall survival: fulvestrant 500 mg vs 250 mg in the randomized CONFIRM trial. J. Natl. Cancer Inst..

[5] Thurlimann B, Robertson JF, Nabholtz JM, Buzdar A, Bonneterre J, Arimidex Study Group (2003). Efficacy of tamoxifen following anastrozole (‘Arimidex’) compared with anastrozole following tamoxifen as first-line treatment for advanced breast cancer in postmenopausal women. Eur. J. Cancer.

[6] Yardley DA, Noguchi S, Pritchard KI, Burris HA, Baselga J, Gnant M (2013). Everolimus plus exemestane in postmenopausal patients with HR(+) breast cancer: BOLERO-2 final progression-free survival analysis. Adv. Ther..

[7] Ghiringhelli F, Menard C, Terme M, Flament C, Taieb J, Chaput N (2005). CD4+CD25+ regulatory T cells inhibit natural killer cell functions in a transforming growth factor-beta-dependent manner. J. Exp. Med..

[8] Ghiringhelli F, Larmonier N, Schmitt E, Parcellier A, Cathelin D, Garrido C (2004). CD4+CD25+ regulatory T cells suppress tumor immunity but are sensitive to cyclophosphamide which allows immunotherapy of established tumors to be curative. Eur. J. Immunol..

[9] Colleoni M, Rocca A, Sandri MT, Zorzino L, Masci G, Nole F (2002). Low-dose oral methotrexate and cyclophosphamide in metastatic breast cancer: antitumor activity and correlation with vascular endothelial growth factor levels. Ann Oncol.

[10] Orlando L, Cardillo A, Rocca A, Balduzzi A, Ghisini R, Peruzzotti G (2006). Prolonged clinical benefit with metronomic chemotherapy in patients with metastatic breast cancer. Anticancer Drugs.

[11] Dellapasqua S, Bertolini F, Bagnardi V, Campagnoli E, Scarano E, Torrisi R (2008). Metronomic cyclophosphamide and capecitabine combined with bevacizumab in advanced breast cancer. J. Clin. Oncol..

[12] Wong NS, Buckman RA, Clemons M, Verma S, Dent S, Trudeau ME (2010). Phase I/II trial of metronomic chemotherapy with daily dalteparin and cyclophosphamide, twice-weekly methotrexate, and daily prednisone as therapy for metastatic breast cancer using vascular endothelial growth factor and soluble vascular endothelial growth factor receptor levels as markers of response. J. Clin. Oncol..

[13] Savas P, Salgado R, Denkert C, Sotiriou C, Darcy PK, Smyth MJ (2016). Clinical relevance of host immunity in breast cancer: from TILs to the clinic. Nat. Rev. Clin. Oncol..

[14] Li Q, Liu X, Wang D, Wang Y, Lu H, Wen S (2020). Prognostic value of tertiary lymphoid structure and tumour infiltrating lymphocytes in oral squamous cell carcinoma. Int. J. Oral Sci..

[15] Mahmoud SM, Paish EC, Powe DG, Macmillan RD, Grainge MJ, Lee AH (2011). Tumor-infiltrating CD8+ lymphocytes predict clinical outcome in breast cancer. J. Clin. Oncol..

[16] Martinet L, Garrido I, Filleron T, Le Guellec S, Bellard E, Fournie JJ (2011). Human solid tumors contain high endothelial venules: Association with T- and B-lymphocyte infiltration and favorable prognosis in breast cancer. Cancer Res..

[17] Lee HJ, Kim JY, Park IA, Song IH, Yu JH, Ahn JH (2015). Prognostic significance of tumor-infiltrating lymphocytes and the tertiary lymphoid structures in HER2-positive breast cancer treated with adjuvant trastuzumab. Am. J. Clin. Pathol..

[18] Dieci MV, Radosevic-Robin N, Fineberg S, van den Eynden G, Ternes N, Penault-Llorca F (2018). Update on tumor-infiltrating lymphocytes (TILs) in breast cancer, including recommendations to assess TILs in residual disease after neoadjuvant therapy and in carcinoma in situ: A report of the International Immuno-Oncology Biomarker Working Group on Breast Cancer. Semin. Cancer Biol..

[19] Acar E, Esendagli G, Yazici O, Dursun A (2022). Tumor-infiltrating lymphocytes (TIL), tertiary lymphoid structures (TLS), and expression of PD-1, TIM-3, LAG-3 on TIL in invasive and in situ ductal breast carcinomas and their relationship with prognostic factors. Clin. Breast Cancer.

[20] Solinas C, Garaud S, De Silva P, Boisson A, Van den Eynden G, de Wind A (2017). Immune checkpoint molecules on tumor-infiltrating lymphocytes and their association with tertiary lymphoid structures in human breast cancer. Front. Immunol..

[21] Wang J, Xu B, Wang W, Zhai X, Chen X (2018). Efficacy and safety of fulvestrant in postmenopausal patients with hormone receptor-positive advanced breast cancer: A systematic literature review and meta-analysis. Breast Cancer Res. Treat..

[22] Conley, C. C., Bishop, B. T., Andersen, B. L. Emotions and emotion regulation in breast cancer survivorship. *Healthcare (Basel)* **4** (2016). 10.3390/healthcare4030056 PMC5041057 27517969

[23] Shiiki S, Sonoo H, Seki M, Nomura T, Hironou M, Ookubo S (2006). Therapeutic efficacy of capecitabine on advanced and recurrent breast cancer with special reference to time to progression. Gan To Kagaku Ryoho.

[24] Mlineritsch B, Tausch C, Singer C, Luschin-Ebengreuth G, Jakesz R, Ploner F (2008). Exemestane as primary systemic treatment for hormone receptor positive post-menopausal breast cancer patients: A phase II trial of the Austrian Breast and Colorectal Cancer Study Group (ABCSG-17). Breast Cancer Res Treat.

[25] Livshits Z, Rao RB, Smith SW (2014). An approach to chemotherapy-associated toxicity. Emerg. Med. Clin. N. Am..

[26] Finn RS, Martin M, Rugo HS, Jones S, Im SA, Gelmon K (2016). Palbociclib and letrozole in advanced breast cancer. N. Engl. J. Med..

[27] Lei W, Li H, Song G, Zhang R, Ran R, Yan Y (2020). Efficacy and safety of fulvestrant 500mg in hormone-receptor positive human epidermal receptor 2 negative advanced breast cancer: A real-world study in China. J. Cancer.

[28] Ahlmann M, Hempel G (2016). The effect of cyclophosphamide on the immune system: Implications for clinical cancer therapy. Cancer Chemother. Pharmacol..

[29] Zhao, Y. *et al*. Combination of DC/CIK adoptive T cell immunotherapy with chemotherapy in advanced non-small-cell lung cancer (NSCLC) patients: A prospective patients’ preference-based study (PPPS). *Clin. Transl. Oncol.* (2018). 10.1007/s12094-018-1968-3 30374838

[30] Qiao G, Wang X, Zhou L, Zhou X, Song Y, Wang S (2019). Autologous dendritic cell-cytokine induced killer cell immunotherapy combined with S-1 plus cisplatin in patients with advanced gastric cancer: A prospective study. Clin. Cancer Res..

[31] Jiang N, Qiao G, Wang X, Morse MA, Gwin WR, Zhou L (2017). Dendritic cell/cytokine-induced killer cell immunotherapy combined with S-1 in patients with advanced pancreatic cancer: A prospective study. Clin. Cancer Res..

[32] Song QK, Ren J, Zhou XN, Wang XL, Song GH, Di LJ (2015). The prognostic value of peripheral CD4+CD25+ T lymphocytes among early stage and triple negative breast cancer patients receiving dendritic cells-cytokine induced killer cells infusion. Oncotarget.

[33] Baecher-Allan C, Wolf E, Hafler DA (2005). Functional analysis of highly defined, FACS-isolated populations of human regulatory CD4+ CD25+ T cells. Clin. Immunol..

[34] Vignali DA, Collison LW, Workman CJ (2008). How regulatory T cells work. Nat. Rev. Immunol..

[35] Wolf AM, Wolf D, Steurer M, Gastl G, Gunsilius E, Grubeck-Loebenstein B (2003). Increase of regulatory T cells in the peripheral blood of cancer patients. Clin. Cancer Res..

[36] Bates GJ, Fox SB, Han C, Leek RD, Garcia JF, Harris AL (2006). Quantification of regulatory T cells enables the identification of high-risk breast cancer patients and those at risk of late relapse. J. Clin. Oncol..

[37] Akbari A, Rezaei A (2012). In vitro selective depletion of CD4(+)CD25(+) regulatory T-cells from PBMC using anti-tac-SAP. J. Immunotoxicol..

[38] Gamcsik MP, Dolan ME, Andersson BS, Murray D (1999). Mechanisms of resistance to the toxicity of cyclophosphamide. Curr. Pharm. Des..

[39] Huang B, Zhao J, Lei Z, Shen S, Li D, Shen GX (2009). miR-142-3p restricts cAMP production in CD4+CD25- T cells and CD4+CD25+ TREG cells by targeting AC9 mRNA. EMBO Rep..

[40] Sautes-Fridman C, Petitprez F, Calderaro J, Fridman WH (2019). Tertiary lymphoid structures in the era of cancer immunotherapy. Nat. Rev. Cancer.

[41] Schumacher TN, Thommen DS (2022). Tertiary lymphoid structures in cancer. Science.

[42] Wang B, Liu J, Han Y, Deng Y, Li J, Jiang Y (2022). The presence of tertiary lymphoid structures provides new insight into the clinicopathological features and prognosis of patients with breast cancer. Front. Immunol..

[43] Barb, A. C. *et al*. Tertiary lymphoid structures (TLSs) and stromal blood vessels have significant and heterogeneous impact on recurrence, lymphovascular and perineural invasion amongst breast cancer molecular subtypes. *Cells* **12** (2023). 10.3390/cells12081176 PMC10136904 37190085

[44] Lutz ER, Wu AA, Bigelow E, Sharma R, Mo G, Soares K (2014). Immunotherapy converts nonimmunogenic pancreatic tumors into immunogenic foci of immune regulation. Cancer Immunol. Res..

[45] Goc J, Germain C, Vo-Bourgais TK, Lupo A, Klein C, Knockaert S (2014). Dendritic cells in tumor-associated tertiary lymphoid structures signal a Th1 cytotoxic immune contexture and license the positive prognostic value of infiltrating CD8+ T cells. Cancer Res..

[46] Ge Y, Domschke C, Stoiber N, Schott S, Heil J, Rom J (2012). Metronomic cyclophosphamide treatment in metastasized breast cancer patients: immunological effects and clinical outcome. Cancer Immunol. Immunother..

[47] Horeweg N, Workel HH, Loiero D, Church DN, Vermij L, Leon-Castillo A (2022). Tertiary lymphoid structures critical for prognosis in endometrial cancer patients. Nat. Commun..

[48] Posch F, Silina K, Leibl S, Mundlein A, Moch H, Siebenhuner A (2018). Maturation of tertiary lymphoid structures and recurrence of stage II and III colorectal cancer. Oncoimmunology.

