# Supplementary material for: Combined oral low-dose cyclophosphamide endocrine therapy may improve clinical response among patients with metastatic breast cancer via Tregs in TLSs
Source: Sci Rep. 2024 Jun 11;14:13432. doi: 10.1038/s41598-024-64042-3 (PMC11166640; doi:10.1038/s41598-024-64042-3)

**Supplementary table 1: Patients’ ages.**

| **CY + ET (Age)** | **ET(Age)** |
| --- | --- |
| 65 | 45 |
| 66 | 46 |
| 67 | 50 |
| 78 | 59 |
| 79 | 49 |
| 65 | 52 |
| 79 | 51 |
| 66 | 54 |
| 65 | 45 |
| 53 | 44 |
| 65 | 53 |
| 66 | 46 |
| 67 | 44 |
| 65 | 61 |
| 66 | 59 |
| 77 | 60 |
| 65 | 60 |
| 66 | 61 |
| 65 | 66 |
| 66 | 67 |
| 67 | 66 |
| 66 | 68 |
| 65 | 66 |
| 67 | 65 |
| 66 | 65 |
| 65 | 66 |
| 65 | 65 |
| 65 | 65 |
| 66 | 66 |
| 54 | 65 |
| 53 | 66 |
| 65 | 67 |
| 65 | 65 |
| 67 | 65 |
| 58 | 65 |
| 67 | 66 |
| 65 | 67 |
| 66 | 67 |
| 49 | 66 |
| 65 | 66 |
| 55 | 65 |
| 65 | 66 |
| 56 | 65 |
| 65 | 66 |
| 67 | 67 |
| 66 | 66 |
| 65 | 65 |
| 66 | 68 |
| 67 | 69 |
| 68 | 66 |
| 65 |  |
| 66 |  |
| 52 |  |
| 66 |  |
| 65 |  |
| 52 |  |
| 66 |  |
| 60 |  |
| 59 |  |
| 66 |  |
| 59 |  |
| 64 |  |
| 48 |  |
| 54 |  |
| 58 |  |
| 57 |  |
| 51 |  |
| 60 |  |
| 65 |  |
| 52 |  |
| 66 |  |
| 49 |  |
| 63 |  |
| 64 |  |
| 63 |  |
| 58 |  |
| 55 |  |
| 66 |  |
| 53 |  |
| 66 |  |
| 67 |  |
| 65 |  |
| 56 |  |
| 53 |  |
| 66 |  |

**Supplementary table 2: The Treg values before and after CY+ET treatment.**

|  | **CY+ET (CR+PR+SD)** | **CY + ET**  **(PD)** | |
| --- | --- | --- | --- |
| **pre-**  **CD4+CD25+** | **post-**  **CD4+CD25+** | **pre-CD4+CD25+** | **post-CD4+CD25+** |
| 1.2 | 0.6 | 3.23 | 3.43 |
| 8 | 2.7 | 1.62 | 2.96 |
| 1.6 | 0.3 | 1.55 | 3.67 |
| 2.1 | 0.72 | 2.19 | 1.17 |
| 4.1 | 0.92 | 1.87 | 4.4 |
| 0.57 | 0.27 | 2.1 | 1.3 |
| 2.27 | 1.17 | 1.54 | 1.26 |
| 2.17 | 0.8 | 1.4 | 1.54 |
| 1.14 | 0.87 | 1.5 | 1.63 |
| 1.31 | 0.9 | 2.5 | 2.37 |
| 2.14 | 1.2 | 1.62 | 1.59 |
| 2.27 | 0.8 | 1.73 | 1.99 |
| 1.24 | 0.6 | 1.58 | 1.64 |
| 2.82 | 1.9 | 1.6 | 1.91 |
| 1.76 | 0.9 | 4.5 | 6.2 |
| 3.19 | 1.76 | 2.9 | 4.52 |
| 2.98 | 1.76 | 1.78 | 1.97 |
| 3.16 | 1.7 | 2.79 | 4.21 |
| 1.2 | 0.6 | 1.12 | 1.97 |
| 8 | 2.7 | 2.67 | 3.78 |
| 1.6 | 0.3 | 1.88 | 1.91 |
| 2.1 | 0.72 | 1.95 | 2.21 |
| 4.1 | 0.92 |  |  |
| 0.57 | 0.27 |  |  |
| 2.27 | 1.17 |  |  |
| 2.17 | 0.8 |  |  |
| 1.14 | 0.87 |  |  |
| 1.31 | 0.9 |  |  |
| 2.14 | 1.2 |  |  |
| 2.27 | 0.8 |  |  |
| 1.24 | 0.6 |  |  |
| 2.82 | 1.9 |  |  |
| 1.76 | 0.9 |  |  |
| 3.19 | 1.76 |  |  |
| 2.98 | 1.76 |  |  |
| 3.16 | 1.7 |  |  |
| 3.23 | 1.98 |  |  |
| 2.72 | 1.76 |  |  |
| 4.72 | 0.89 |  |  |
| 2.17 | 1.52 |  |  |
| 5.76 | 1.23 |  |  |
| 3.29 | 1.86 |  |  |
| 2.88 | 1.76 |  |  |
| 5.23 | 2.34 |  |  |
| 2.36 | 1.68 |  |  |
| 1.23 | 0.56 |  |  |
| 1.93 | 0.45 |  |  |
| 2.67 | 1.63 |  |  |
| 1.86 | 0.72 |  |  |
| 2.25 | 1.12 |  |  |
| 3.69 | 0.56 |  |  |
| 1.34 | 0.56 |  |  |
| 1.59 | 0.36 |  |  |
| 2.45 | 3.02 |  |  |
| 1.22 | 0.65 |  |  |
| 3.13 | 2.04 |  |  |
| 2.37 | 1.53 |  |  |
| 1.39 | 1.46 |  |  |
| 2.15 | 1.17 |  |  |
| 2.97 | 1.63 |  |  |
| 2.15 | 1.12 |  |  |
| 4.69 | 1.56 |  |  |
| 2.24 | 1.73 |  |  |
| 1.93 | 0.85 |  |  |
| 2.38 | 1.96 |  |  |
| 2.18 | 1.72 |  |  |
| 5.39 | 1.26 |  |  |
| 1.83 | 0.96 |  |  |
| 2.97 | 1.62 |  |  |
| 3.73 | 1.54 |  |  |
| 2.66 | 1.23 |  |  |
| 2.08 | 1.62 |  |  |
| 1.29 | 0.66 |  |  |
| 3.64 | 1.57 |  |  |
| 1.43 | 1.77 |  |  |
| 2.54 | 1.78 |  |  |
| 1.59 | 1.96 |  |  |
| 2.35 | 1.57 |  |  |
| 1.79 | 1.8 |  |  |


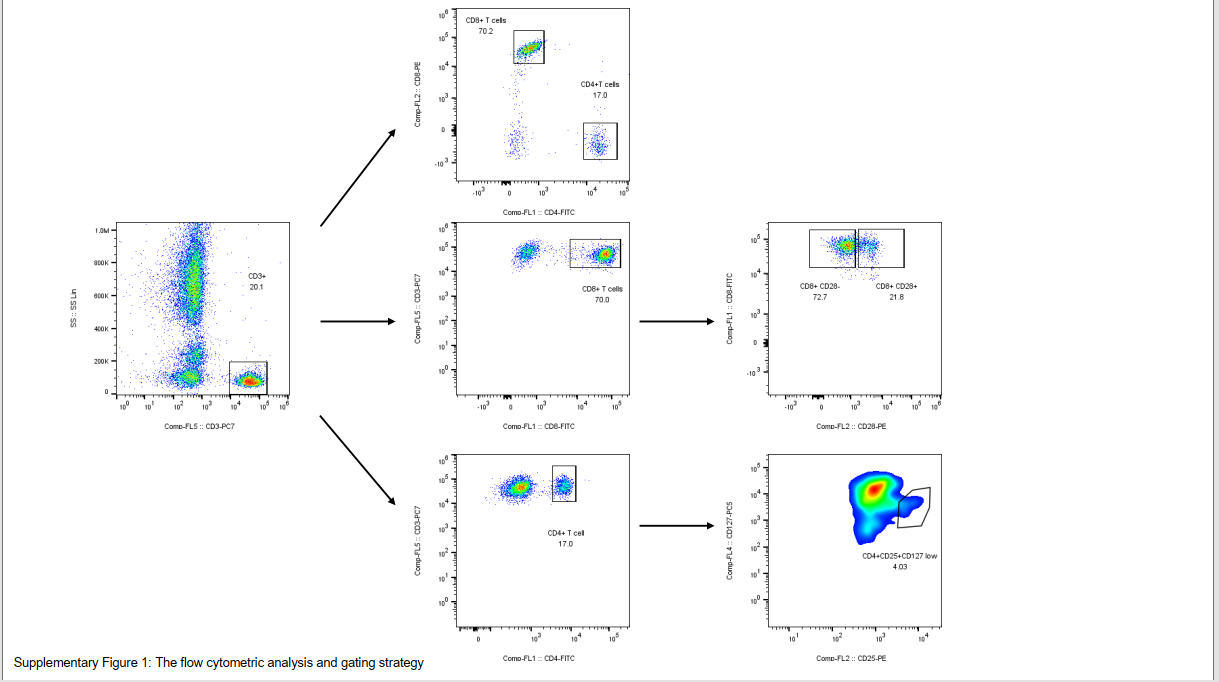


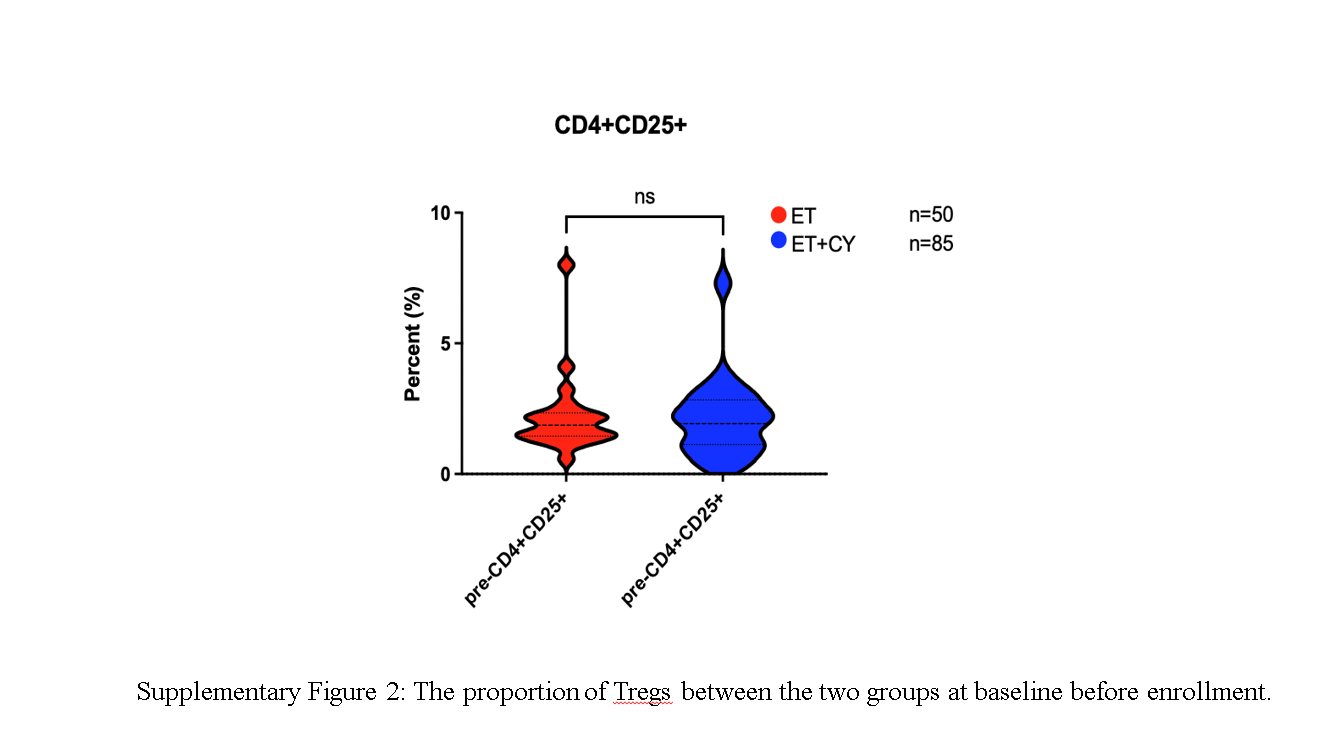

Supplement: Supplementary file 1 — Supplementary Information. [file 41598_2024_64042_MOESM1_ESM.docx]
